# Monitorization of pH and OTR using a multiple shake flask platform: A tool for metabolism and cell growth assessment in mammalian cell cultures

**DOI:** 10.1186/1753-6561-9-S9-P32

**Published:** 2015-12-14

**Authors:** Leticia Liste-Calleja, Tibor Anderlei, Jonatan López-Repullo, Adrián Urbano, Martí Lecina, Jordi J Cairó

**Affiliations:** 1 Chemical Engineering Department, EE, Universitat Autònoma de Barcelona, Spain; 2 Adolf Kühner AG, Birsfelden, Switzerland

## Background

From 1989 the number of bioprocesses based on mammalian cell systems has been continuously increasing from 33% of the total number of new drug approvals (up to 1989) to 60% of that number (period 2010-2014)[1].Due to the high costs of these bioprocesses and the increasing competitiveness in the field, the good characterization and optimization of cell growth and product obtention at laboratory scale have become highly necessary. Moreover, PAT (Process Analytical Technology) initiative has beenspreadingalongthegrowingbiopharmaceuticalindustrysince 2004, when FDA published PAT guidance in order to encourageinnovativepharmaceuticaldevelopment and manufacturing[2]. In this line of thinking, big efforts are directed to develop systems at laboratory scale to obtain real-time cell culture monitoring in a non-invasive manner to avoid cell culture disturbance.

In the present communication, the shake flask reader (SFR) from Presens was used in combination with the RAMOS-System[3] adapted to disposable shake flask to validate it as an useful tool for online monitoring. In this sense, we performed a study of different metabolic behavior of HEK293 cells triggered by means of environmental conditions manipulation. pH and pO2 were monitored through all cell culture and OTR, CTR and RQ were determined. The comparison of those measurements with off-line metabolite and cell growth assessment, along with further analysis of the variables, led to (1) determine a good correlation between pH evolution and HEK293 metabolic behaviour, (2) define an OTR profile corresponding to cell growth evolution and cell activity and (3) get information of cell culture differences under distinct physicochemical circumstances.

## Materials and Methods

HEK293 cell batch cultures were performed in 250mL disposable shake flasks attached to the RAMOS-System and to SFR to get on-line measurements of pH and dissolved oxygen (DO). An initial batch culture with SFMTransFx-293 media 5%FBS and 10%CB5 (80g/L) supplemented was performed. This media has been already tested for the obtention of high cell density cultures (up to 18e6 cell/mL)[4]. Then, two media modifications were carried out in order to grow the cells in a non-desired environment and detect differences-if any- on pH evolution, OTR profile, cell growth, glucose consumption, lactate production and GFP production. These modifications consisted on: (1) acidification of media to pH = 6.6 by means of HCl addition and (2) acidification of media to pH = 6.6 (HCl addition) and 12mM sodium lactate addition.

## Results

**Figure 1:**
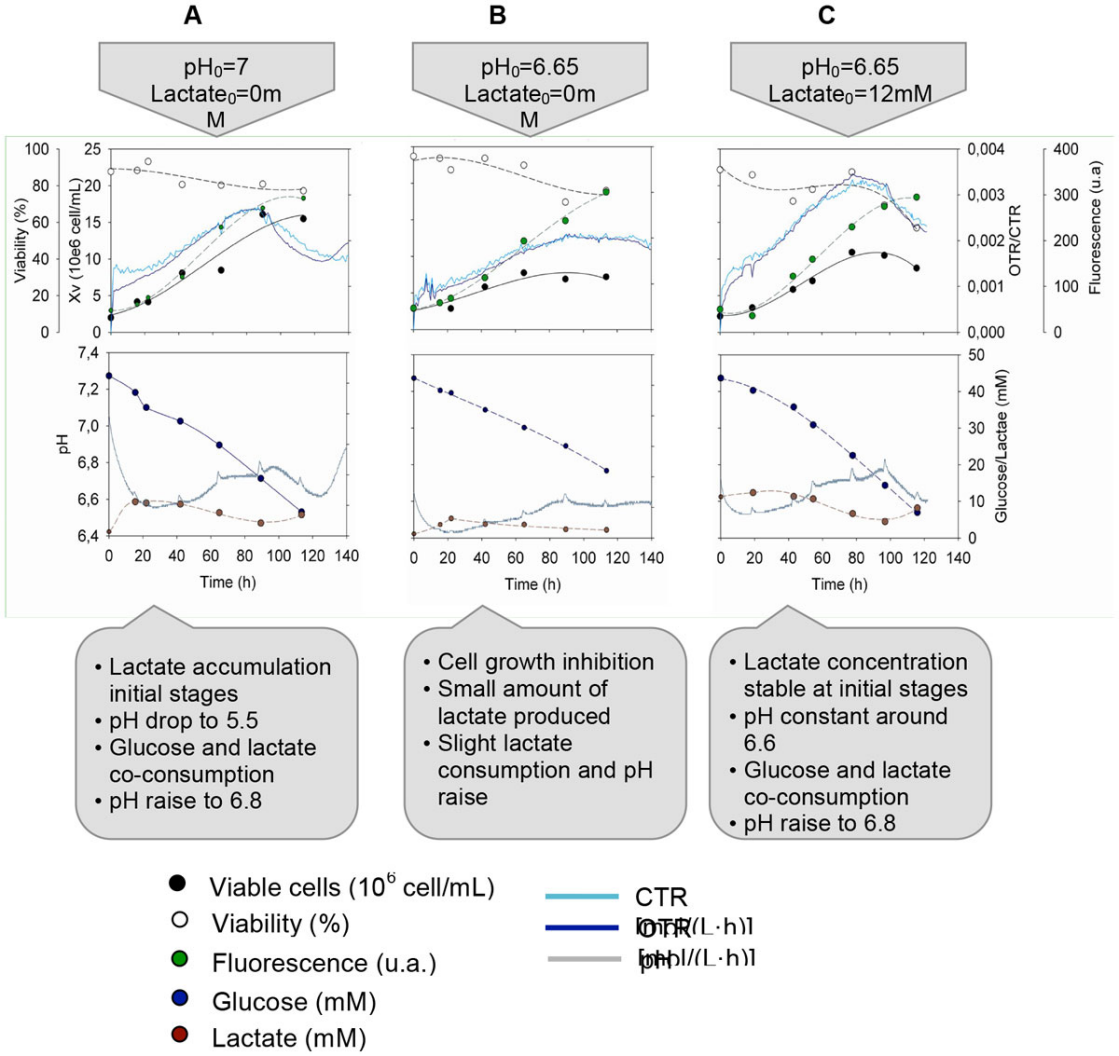
**Off-line profiles (growth, viability, fluorescence, glucose and lactate) and on-line parameters (pH, OTR, CTR) of HEK293 cell cultures under different environment conditions**.

When sodium lactate was not added to media at low pH (Figure 1B), cell growth inhibition was detected resulting in a decrement of μmaxin comparison to cell cultures in which sodium lactate was added (Figure 1C) and also to control cultures (Figure 1A). Therefore, a significant decrease on cell expansion was observed reaching values about 9·106cell/mL, or in other words, a 50% drop on Xvmax. Interestingly, with the simple addition of sodium lactate the expected cell growth inhibition due to low pH was completely avoided. For the three conditions tested, it was found out that OTR profile perfectly fitted cell growth during exponential phase. In addition, a decrement of OTR slope was detected before a viable cell number drop was noticed. Accordingly, the linearization of OTR values showed shorter linear phase in comparison to the viable number cells linearization.

Concerning pH monitoring, it could be assessed that the profiles correlated good with lactate evolution. Lactate secretion was completely suppressed when lactate was added to cell media at pH 6.65 and lactic uptake started from the initial stages of cell culture. Consequently, media pH was maintained constant for approximately 20h and thereafter, it started to increase. In contrast, when lactate was not added at pH0 = 6.65 an initial secretion of lactate was detected during the lag phase (ΔLac ≈ 4mM) and pH dropped accordingly. Then, co-metabolism of lactate and glucose was triggered but at lower rates than for cultures were lactate was present from t = 0h. The lower consumption rate of cultures at pH0 = 6.65 and [lactate]0 = 0mM was in good relation with the lower increment of pH value in comparison to the other cell culture conditions.

## Conclusions

Taken into account all the reported results, RAMOS-System in combination with SFR is an instrument with high potential for mammalian cell culture characterization as it provides reliable data of cell metabolism, growth and state. Furthermore, all this data is taken on line in a non-invasive manner and offers continuous measurements. Altogether would meet GMP constraints of a given bioprocess.

## Acknowledgments

The authors would like to thank Kuhner S.A. for providing the equipment. We also would like to thank Dr. A. Kamen (National Research Council of Canada) for kindly providing HEK293 cells, which this work was performed with.

This research was supported by the BIO2012-32151 project from MINECO (Spanish Government).
